# Supplementary material for: ETVs dictate hPSC differentiation by tuning biophysical properties
Source: Nat Commun. 2025 Feb 26;16:1999. doi: 10.1038/s41467-025-56591-6 (PMC11865489; doi:10.1038/s41467-025-56591-6)

Supplementary Figures

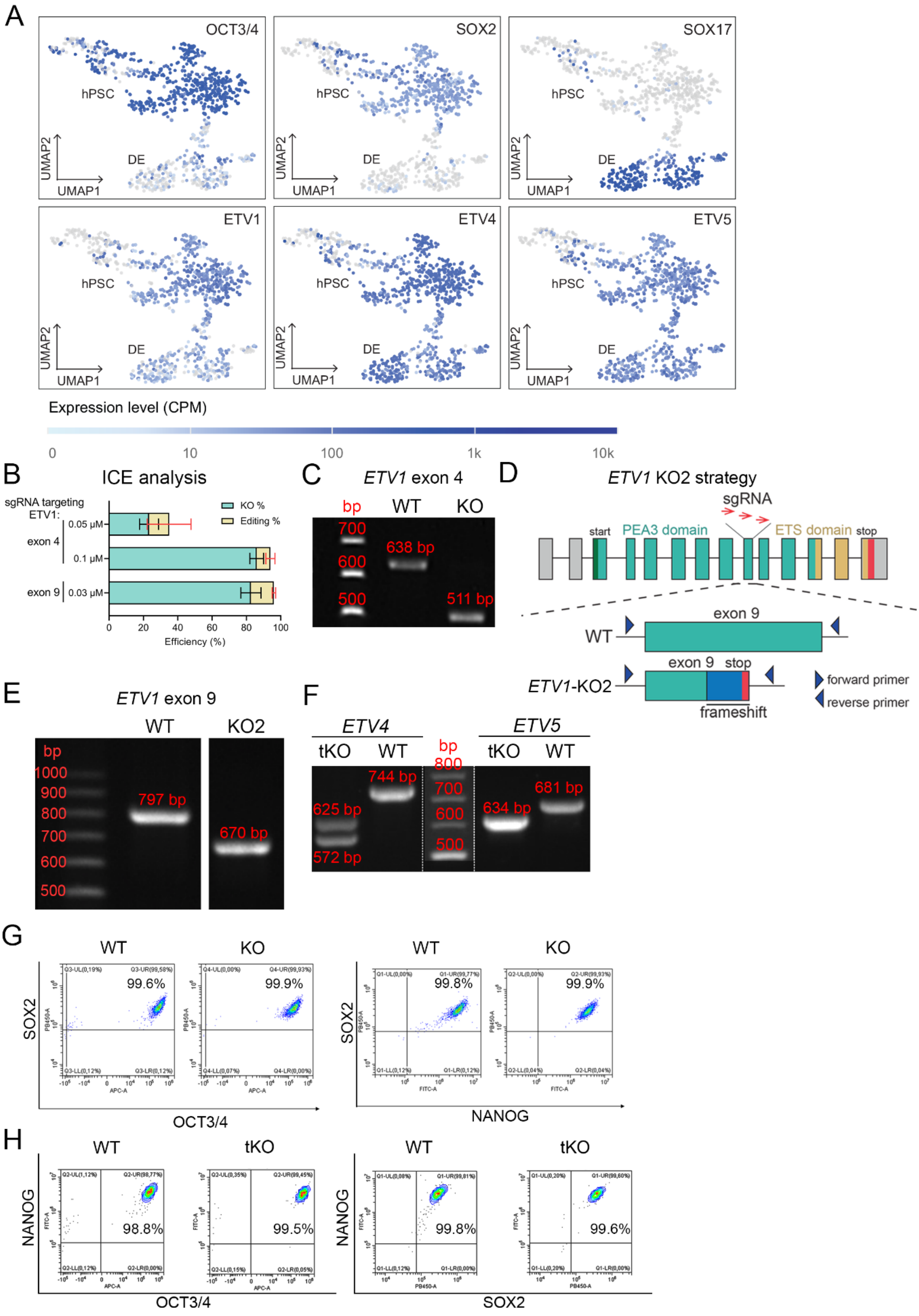

Supplementary Figure 1. Generation of the second loss-of-function *ETV1 KO* hPSC line and preliminary characterization of *ETV1, ETV4, ETV5 KO* hPSCs

A. UMAP plots show cells at the pluripotent stage (OCT3/4 and SOX2 positive) and definitive endoderm (SOX17 positive) (upper panel) expressing *ETV1*, *ETV4*, and *ETV5* (bottom panel). The expression of *ETV1* is higher in hPSCs, while *ETV4* and *ETV5* show comparable expression levels in both hPSCs and DE[1]. Dark blue dots – cells with high gene expression levels; light blue dots – cells with low gene expressions; gray dots– expression not detected. CPM - counts-per-million normalization.

B. The editing efficiency of *ETV1*, as determined by Inference of CRISPR Edits (ICE) analysis, shows an average indel rate at ~80% that caused knock-out mutation (KO%) of *ETV1*. This efficiency was achieved using 0.1 µM sgRNAs targeting exon 4 and 0.03 µM sgRNAs targeting exon 9.

C. Representative picture of the agarose gel electrophoresis demonstrating a 127 bp deletion in exon 4 of the *ETV1* gene in ETV1 KO hPSCs.

D. Strategy to knockout the *ETV1* gene (KO2) in hPSCs using CRISPR/Cas9 approach. Three different sgRNAs (red arrows) targeting exon 9 of the *ETV1* gene were co-transfected into hPSCs, pretreated with doxycycline to induce Cas9 expression. CRISPR/Cas9 edition resulted in a 127 nucleotide deletion and the occurrence of a premature stop codon. Untranslated regions (grey), PEA3 domain (turquoise green), ETS domain (dark yellow), STOP codon (red). Primer positions are indicated by arrowheads.

E. Representative picture of the agarose gel electrophoresis demonstrating a 127 bp deletion in exon 9 of the *ETV1* gene in *ETV1* KO2 hPSCs.

F. Representative picture of agarose gel electrophoresis demonstrating a 119 bp deletion in exon 2/3 of the *ETV4* gene (left panel), and a 47 bp deletion in exon 3/4 of the *ETV5* gene (right panel), in tKO hPSCs.

G. Representative flow cytometry analysis demonstrates similar protein levels of SOX2, OCT3/4 and NANOG in both KO and WT hPSCs, $N = 3$ biological replicates.

H. Representative flow cytometry analysis demonstrates a similar protein levels of SOX2, OCT3/4 and NANOG in both tKO and WT hPSCs, $N = 3$ biological replicates.

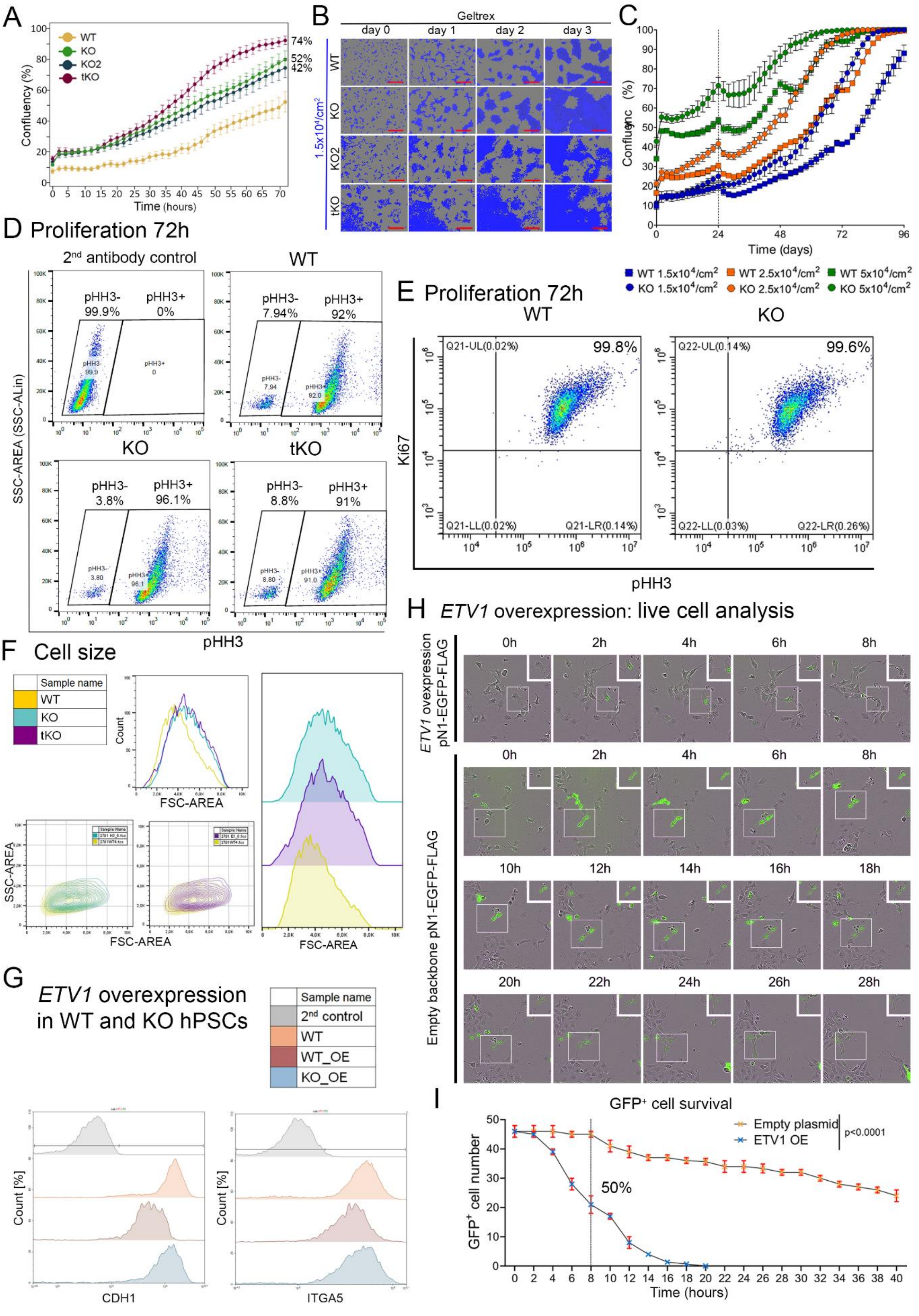

A
WT
KO
KO2
tKO
74%
52%
42%
Confluency (%)
Time (hours)
B
Geltrex
day 0
day 1
day 2
day 3
1.5x10⁴/cm²
C
Confluency (%)
Time (days)
WT 1.5x10⁴/cm²
KO 1.5x10⁴/cm²
WT 2.5x10⁴/cm²
KO 2.5x10⁴/cm²
WT 5x10⁴/cm²
KO 5x10⁴/cm²
D Proliferation 72h
2nd antibody control
pHH3- 99.9%
pHH3+ 0%
WT
pHH3- 7.94%
pHH3+ 92%
KO
pHH3- 3.8%
pHH3+ 96.1%
tKO
pHH3- 8.8%
pHH3+ 91%
SSC-AREA (SSC-ALin)
pHH3
E Proliferation 72h
WT
KO
99.8%
99.6%
Q21-UL(0.02%)
Q21-LL(0.02%)
Q21-LR(0.14%)
Q22-UL(0.14%)
Q22-LL(0.03%)
Q22-LR(0.26%)
Ki67
pHH3
F Cell size
Sample name
WT
KO
tKO
Count
FSC-AREA
SSC-AREA
G
ETV1 overexpression in WT and KO hPSCs
Sample name
2nd control
WT
WT_OE
KO_OE
Count [%]
CDH1
ITGA5
H ETV1 overexpression: live cell analysis
ETV1 overexpression pN1-EGFP-FLAG
Empty backbone pN1-EGFP-FLAG
0h
2h
4h
6h
8h
10h
12h
14h
16h
18h
20h
22h
24h
26h
28h
I
GFP⁺ cell survival
Empty plasmid
ETV1 OE
p<0.0001
50%
GFP⁺ cell number
Time (hours)

Supplementary Figure 2. Increased cell-extracellular matrix and cell-cell adhesion of *ETV1* and *ETV1*/*ETV4*/*ETV5* deficient hPSCs

A. Live-cell confluency analysis of WT (yellow), KO (light green), KO2 (dark green) and tKO (maroon) hPSCs over 70 h of culture shows the highest increase in confluency in case of tKO (74%), with 52% and 42% increases in KO and KO2, respectively, compared to WT. *N* = 3 biological replicates. Scale bar = 400 μm.

B. Representative images of live-cell analysis of WT, KO, KO2. and tKO hPSC on Geltrex-coated plates during a 3-day culture period. All cell lines were seeded at a starting density of 1.5 x 10^5 cells/cm^2. Enhanced confluency in ETV deficient cell lines was observed after 1 day of culture and this trend was maintained till day 3. *N* = 3 biological replicates. Scale bar = 400 μm.

C. Live-cell confluency analysis of KO (circle) and WT (square) hPSCs over 96 h of culture with different starting cell densities: 1.5 x 10^5 cells/cm^2 (blue), 2.5 x 10^5 cells/cm^2 (orange) and 5 x 10^5 cells/cm^2 (green). Increased confluency was observed in KO compared to WT hPSCs independent of the starting cell density. *N* = 3 biological replicates.

D. Representative flow cytometry analysis demonstrates similar proliferation rates (marked by pHH3+ cells) of WT, KO and tKO hPSCs over 72 h culture. *N* = 3 biological replicates.

E. Representative flow cytometry analysis demonstrates similar proliferation levels (marked by Ki67+ cells) of WT and KO hPSCs over 72 h culture. *N* = 3 biological replicates.

F. Representative flow cytometry analysis demonstrates increased cell size (measured as FSC-area) of KO (green) and tKO (maroon) hPSCs compared to WT (yellow) hPSCs. *N* = 3 biological replicates.

G. Representative flow cytometry analysis demonstrates decreased level of adhesion proteins: CDH1 and ITGA5 after doxycycline induction of ETV1 ectopic expression in WT_OE (brown) and KO_OE (blue) hPSC lines compared to WT (orange) hPSCs. CDH1: *N* = 4 biological replicates; ITGA5: *N* = 3 biological replicates.

H. Representative live-cell bright-field images of single hPSCs showing a green fluorescence signal (GFP), following hPSC transfection with either a transient *ETV1* overexpression pN1-EGFP-FLAG or a backbone plasmid as control. The hPSCs with transient ectopic ETV1 expression began detaching from the plate surface at 6 h after GFP signal appearance, while hPSCs transfected with control plasmid continued to grow and maintained GFP expression. *N* = 3 biological replicates.

I. Quantification of GFP+ hPSCs following transfection with *ETV1* cDNA-encoding (ETV1 OE) plasmid or a backbone plasmid as control, demonstrates a continuous decrease in cell number with transient ETV1 OE. The 50% decrease in GFP+ cells is observed at 8 h after the detection of GFP signal, compared to the control. *N* = 4 biological replicates.

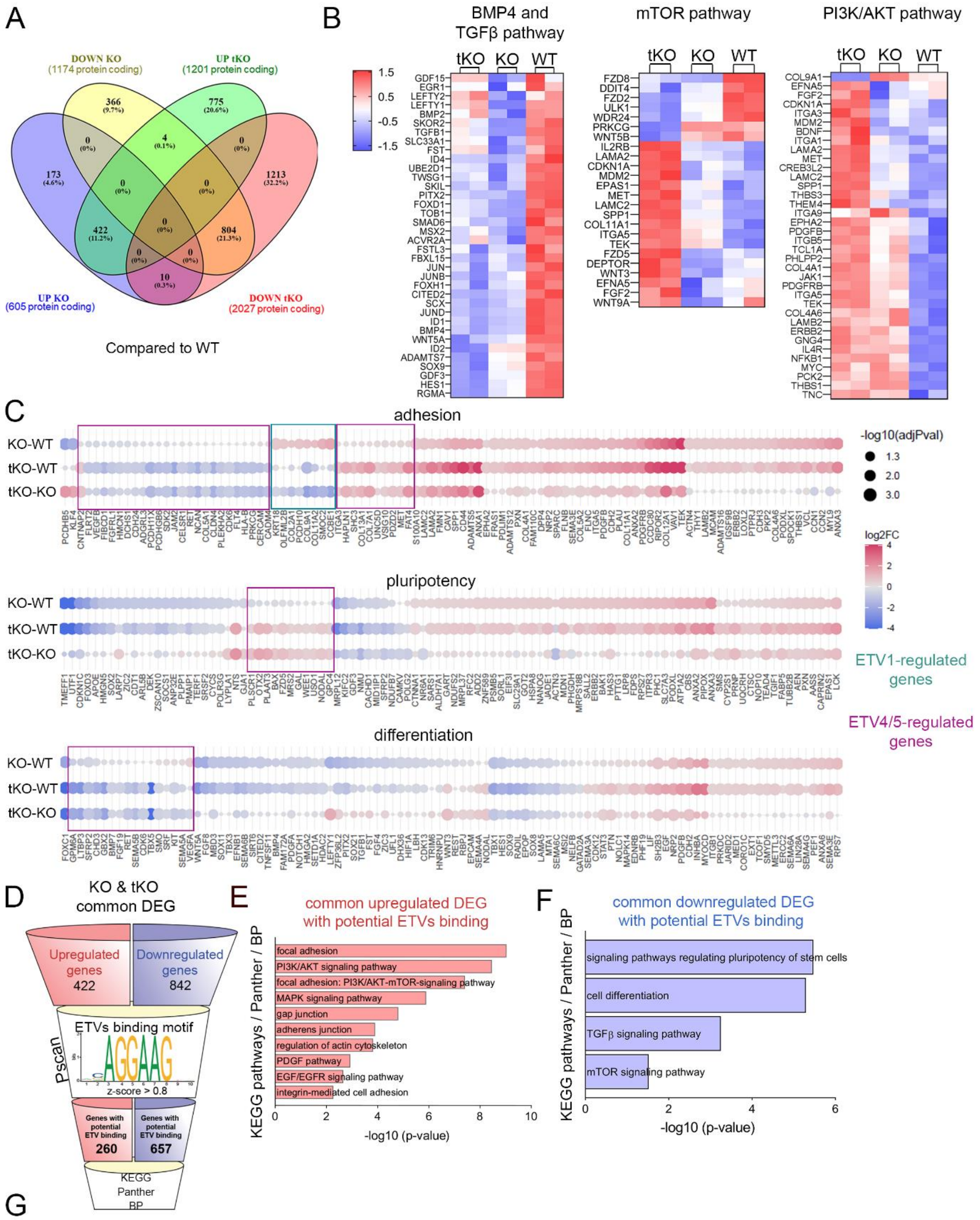

G

Gene	Position from TSS	ETV1 binding sequence	z-score
CDKN1A	-646	CCCGGAAGCA	0.907951
COL4A6	-140	AACGGAAGGT	0.856607
ITGA5	-590	AGCGGAACTT	0.864311
JUN	-539	CCCGGAAGTG	0.928676
PDGFRB	-645	AACGGAAGCC	0.884747
VCL	-222	ACCGGATTCC	0.813414

Supplementary Figure 3. RNA-sequencing analysis reveals genes and pathways differentially expressed in *ETV1* KO and *ETV1*, *ETV4* and *ETV5* tKO hPSCs

A. Venn diagram shows the distribution of individual and common genes with changed expression in KO or tKO compared to WT hPSCs. Significantly upregulated (log2 FC $\geq$ 0.05, $p \leq 0.05$) or downregulated (log2 FC $\leq$ -0.05, $p \leq 0.05$) differentially expressed genes were selected for further analysis.

B. Heatmap shows the expression pattern of differentially expressed genes in KO and tKO associated with TGFβ and BMP, mTOR and PI3K/AKT pathways. Each column represents the studied cell line included in the RNA-seq experiment (WT, KO and tKO in duplicate), while each row represents a corresponding gene. Upregulated genes – red; downregulated genes – blue.

C. Dot plot demonstrates differentially expressed genes associated with adhesion (upper panel), pluripotency (middle panel), and differentiation (bottom panel). ETV1-regulated genes are marked by a green rectangle, while ETV4/ETV5-regulated genes are marked by maroon rectangles.

D. Scheme presenting the *in silico* analysis of the *ETV1*, *ETV4* and *ETV5* DNA binding motif prediction among differentially expressed genes (common for the KO vs. WT and tKO vs. WT comparisons). Among the 422 upregulated (red) and 842 downregulated (blue) genes in KO and tKO compared to WT, 260 and 657 genes, respectively, contain a potential binding motif for PEA3 subfamily members.

E. Bar plot representing the enriched functional terms related to cell adhesion, PI3K/AKT or adherence junctions (based on KEGG, Panther and Biological Processes databases) among common upregulated genes for KO and tKO compared to WT, with a potential binding site for PEA3 subfamily members.

F. Bar plot representing the enriched functional terms related to pathways associated with pluripotency or differentiation (based on KEGG, Panther and Biological Processes databases), among common downregulated genes for KO and tKO compared to WT, with a potential binding site for PEA3 subfamily members.

G. Table shows the position relative to the transcription start site (TSS), the *ETV1* binding sequence, and z-score for ETV1-dependent genes associated with cell adhesion, i.e. *ITGA5, VCL, PDGFRB,* and *COL4A* or genes regulating pluripotency, i.e. *JUN* and *CDKN1A*.

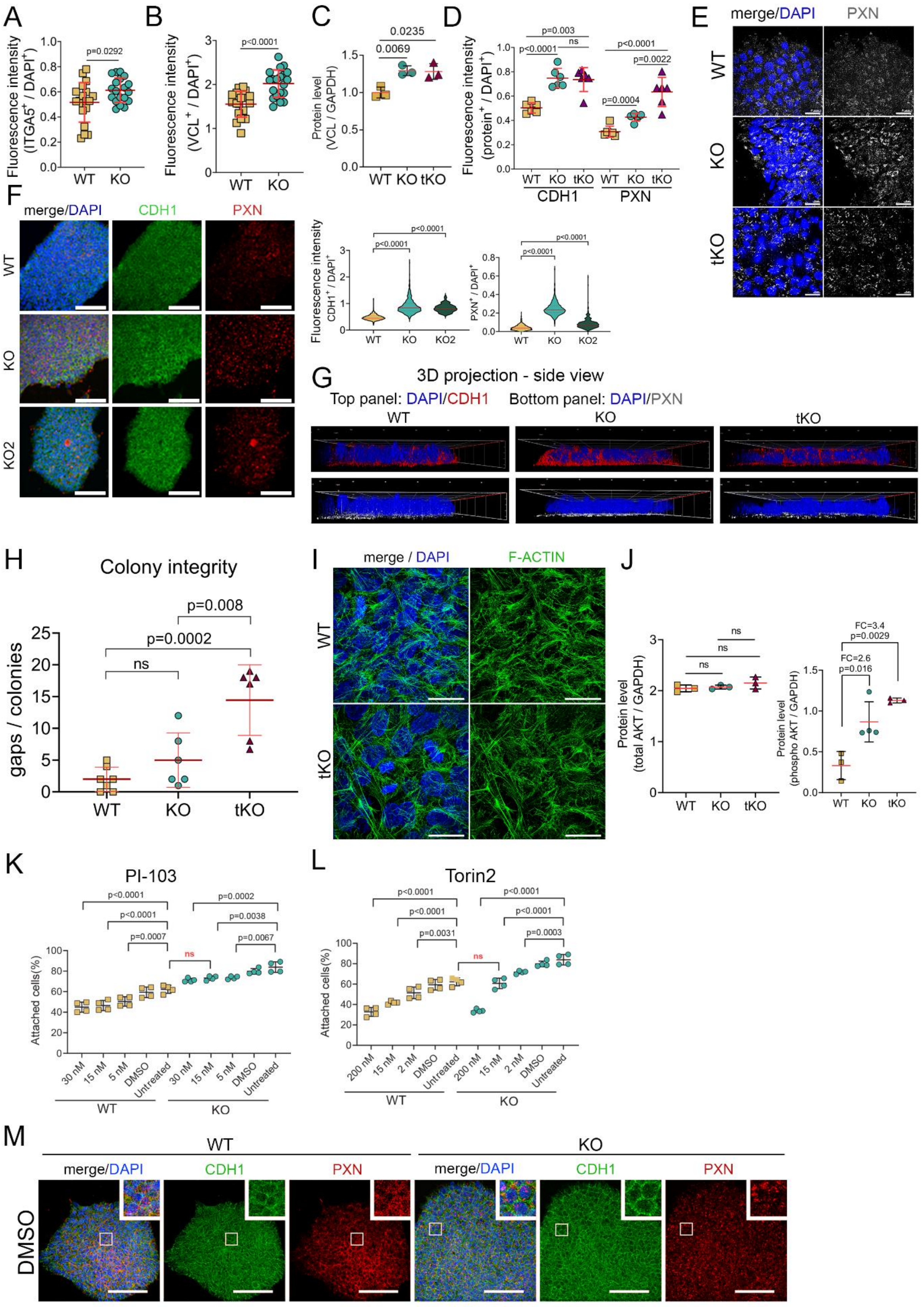
A
Fluorescence intensity (ITGA5+ / DAPI+)
p=0.0292
WT KO
B
Fluorescence intensity (VCL+ / DAPI+)
p<0.0001
WT KO
C
Protein level (VCL / GAPDH)
0.0235
0.0069
WT KO tKO
D
Fluorescence intensity (protein+ / DAPI+)
p=0.003
p<0.0001
ns
p<0.0001
p=0.0022
p=0.0004
WT KO tKO WT KO tKO
CDH1 PXN
E
merge/DAPI PXN
WT
KO
tKO
F
merge/DAPI CDH1 PXN
WT
KO
KO2
Fluorescence intensity
CDH1+ / DAPI+
PXN+ / DAPI+
p<0.0001
p<0.0001
WT KO KO2
G
3D projection - side view
Top panel: DAPI/CDH1 Bottom panel: DAPI/PXN
WT KO tKO
H
Colony integrity
gaps / colonies
p=0.008
p=0.0002
ns
WT KO tKO
I
merge / DAPI F-ACTIN
WT
tKO
J
Protein level (total AKT / GAPDH)
ns
ns
ns
WT KO tKO
Protein level (phospho AKT / GAPDH)
FC=3.4
p=0.0029
FC=2.6
p=0.016
WT KO tKO
K
PI-103
Attached cells(%)
p<0.0001
p<0.0001
p=0.0007
p=0.0002
p=0.0038
p=0.0067
ns
30 nM 15 nM 5 nM DMSO Untreated 30 nM 15 nM 5 nM DMSO Untreated
WT KO
L
Torin2
Attached cells(%)
p<0.0001
p<0.0001
p=0.0031
p<0.0001
p<0.0001
p=0.0003
ns
200 nM 15 nM 2 nM DMSO Untreated 200 nM 15 nM 2 nM DMSO Untreated
WT KO
M
WT KO
merge/DAPI CDH1 PXN merge/DAPI CDH1 PXN
DMSO

Supplementary Figure 4. Genes regulated by ETV1 in hPSCs are involved in cell adhesion and PI3K/AKT pathway

- **A.** Quantification of fluorescence intensity (ITGA5+/DAPI) calculated from the immunofluorescence images of ITGA5 staining in WT (yellow) and KO (green) hPSCs. *N* = 3 biological replicates.
- **B.** Quantification of fluorescence intensity (VCL+/DAPI) calculated in WT (yellow) and KO (green) hPSCs. *N* = 3 biological replicates.
- **C.** Quantification of western blotting with antibody against VCL (upper plot) in WT, KO, and tKO hPSCs. *N* = 3 biological replicates.
- **D.** Quantification of fluorescence intensity (CDH1+/DAPI and PXN+/DAPI) in WT (yellow), KO (green), and tKO (maroon) hPSCs. *N* = 3 biological replicates.
- **E.** High-resolution microscopy images of PXN (grey) immunofluorescence staining confirming different distributions of PXN protein in WT, KO, and tKO hPSC colonies. Scale bar = 20 nm.
- **F.** Representative immunofluorescence images show increased protein levels of CDH1 (green) and PXN (red) in KO and KO2 compared to WT hPSCs. DAPI marks the nuclei (blue). On the right, quantification of fluorescence intensity (PXN+/DAPI) calculated from the immunofluorescence images of PXN staining in WT (yellow), KO (light green), and KO2 (dark green) hPSCs. *N* = 3 biological replicates. Scale bar = 100 µm.
- **G.** High-resolution microscopy 3D projections (side view) of CDH1 (red, upper panel) and PXN (grey, bottom panel) immunofluorescence staining confirm the highest protein levels of CDH1 in KO, followed by tKO, compared to WT hPSCs. Additionally, the images reveal different distributions of CDH1 and PXN proteins in WT, KO, and tKO hPSC colonies.
- **H.** Quantification of CDH1 immunofluorescence staining shows gaps in the colonies of WT, KO, and tKO hPSCs.
- **I.** High-resolution microscopy images of F-ACTIN (green) immunofluorescence staining show different actin cytoskeleton organization along WT and tKO hPSC at colony edges. Scale bar = 20 nm.
- **J.** Quantification of western blotting with antibody against phospho-AKT (left plot) and total AKT (right plot) proteins confirm increased phosphorylation of AKT protein in KO and tKO compared to WT hPSCs, while total AKT protein level remains unchanged in *ETV1* and *ETV1*/*ETV4*/*ETV5* deficient hPSCs compared to WT. *N* = 3 biological replicates.
- **K.** Quantification of crystal violet staining shows a dose-dependent decrease in a fraction of attached WT (yellow) and KO (green) hPSCs after PI3K/AKT pathway inhibition by PI-103, compared to untreated and control (DMSO-treated) WT or KO hPSCs, respectively. *N* = 4 biological replicates.

L. Quantification of crystal violet staining shows a dose-dependent decrease in a fraction of attached WT (yellow) and KO (green) hPSCs after PI3K/AKT pathway inhibition by Torin2, compared to untreated and control (DMSO-treated) WT or KO hPSCs, respectively. *N* = 4 biological replicates.

M. Representative images of immunofluorescence staining show protein levels of CDH1 (green) and PXN (red) in WT and KO hPSCs cultured in medium with DMSO; a control experimental condition tested alongside the 72-hour PI3K/AKT pathway activity modulation experiments. DAPI marks the nuclei (blue). *N* = 3 biological replicates. Scale bar = 200 μm.

The following tests were used to determine the *p*-values shown on the graph: a two-sided student's t-test for plots **A-B**, the Welch's t-test for plot **C**, a one-way ANOVA with Turkey's correction for multiple comparisons for plot **D**, a one-way ANOVA for multiple comparisons for plots **F**, **H**, and **J-L**. Data are presented as means ± SDs.

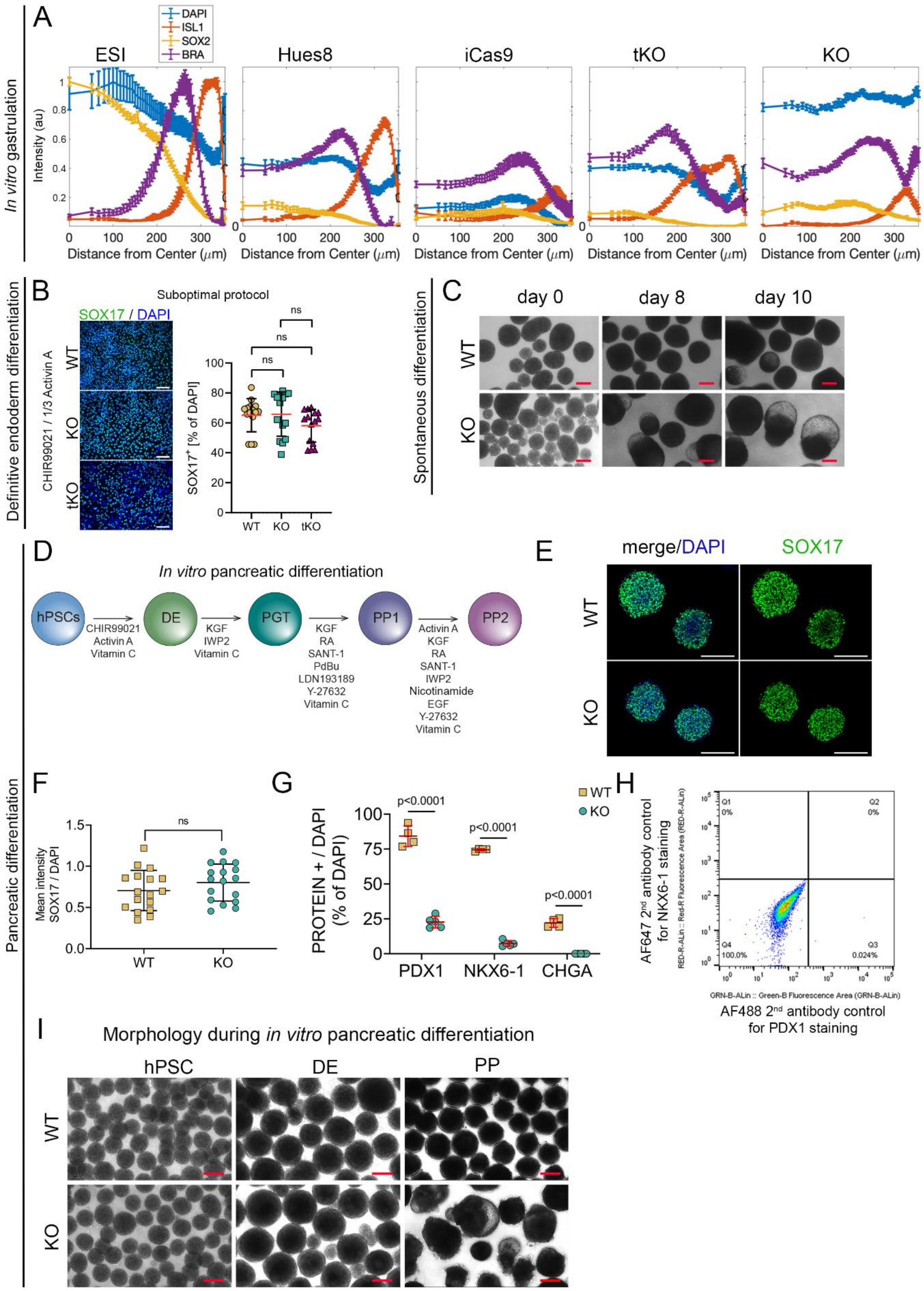
A
In vitro gastrulation
DAPI
ISL1
SOX2
BRA
ESI
Hues8
iCas9
tKO
KO
Intensity (au)
Distance from Center (μm)
B
Definitive endoderm differentiation
Suboptimal protocol
SOX17 / DAPI
CHIR99021 / 1/3 Activin A
WT
KO
tKO
ns
SOX17+ [% of DAPI]
C
Spontaneous differentiation
day 0
day 8
day 10
WT
KO
D
In vitro pancreatic differentiation
hPSCs
DE
PGT
PP1
PP2
CHIR99021
Activin A
Vitamin C
KGF
IWP2
Vitamin C
KGF
RA
SANT-1
PdBu
LDN193189
Y-27632
Vitamin C
Activin A
KGF
RA
SANT-1
IWP2
Nicotinamide
EGF
Y-27632
Vitamin C
E
merge/DAPI
SOX17
WT
KO
Pancreatic differentiation
F
Mean intensity SOX17 / DAPI
ns
WT
KO
G
PROTEIN + / DAPI (% of DAPI)
p<0.0001
p<0.0001
p<0.0001
WT
KO
PDX1
NKX6-1
CHGA
H
AF647 2nd antibody control for NKX6-1 staining
Q1 0%
Q2 0%
Q4 100.0%
Q3 0.024%
AF488 2nd antibody control for PDX1 staining
I
Morphology during in vitro pancreatic differentiation
hPSC
DE
PP
WT
KO

Supplementary Figure 5. Deletions of ETV genes affect *in vitro* gastruloid formation, direction of spontaneous *in vitro* differentiation and pancreatic *in vitro* differentiation

A. Distribution of positive signals from DAPI (blue), ISL1 (red), SOX2 (yellow) and BRA (purple) immunofluorescence staining from the center to the edge of gastruloids was performed in ESI, Hues8, iCas9, tKO, and KO gastruloids. *N* = 3 biological replicates.

B. WT, KO, and tKO differentiation to definitive endoderm using suboptimal protocol with a decreased dose of Activin A.

C. Bright-field images of WT and KO embryoid bodies (EB) during spontaneous *in vitro* differentiation of hPSC, without the addition of lineage-specific growth factors. At day 0, the morphology of WT and KO EBs were similar, while at day 8 and 10, the KO EBs were bigger than WT EBs, and cyst-like structures were observed in KO EBs.

D. Detail scheme showing the timeline of 12-day hPSC *in vitro* differentiation into pancreatic progenitors. During differentiation, hPSCs pass through following stages: definitive endoderm (DE), primitive gut tube (PGT), pancreatic progenitors 1 (PP1), pancreatic progenitors 2 (PP2). The small molecules driving differentiation towards indicated stages are listed below the arrows.

E. Representative immunofluorescence staining of SOX17 (green) shows similar protein levels of SOX17 between KO and WT at the definitive endoderm stage during *in vitro* differentiation. *N* = 3 biological replicates. Scale bar = 200 µm.

F. Quantification of fluorescence intensity (SOX17+/DAPI) calculated from the immunofluorescence images of SOX17 staining of WT (yellow) and KO (green) DE confirmed no difference in SOX17 protein levels between KO and WT cells. *N* = 3 biological replicates.

G. The graph shows the relative fluorescence intensity of PDX1, NKX6-1, and CHGA, normalized to DAPI staining, in WT (yellow) and KO (green) PPs. *N* = 4 biological replicates.

H. Secondary antibody negative control was used to set the gates for flow cytometry analysis for NKX6-1 (vertical) and PDX1 (horizontal) staining at the pancreatic progenitor stage of *in vitro* differentiation.

I. Representative bright-field images of WT and KO cells differentiated to pancreatic progenitors (PPs). The overall morphology of 3D spheres at hPSC and DE does not differ between KO and WT, while at the PP stage, KO spheres are bigger than WT and display cyst-like structures. Scale bar = 200 µm.

For plot **B**, a one-way ANOVA with Turkey's correction for multiple comparisons was used, for plots **F-G**, a two-sided student's t-test was used to determine the *p*-values shown on the graph. Data are presented as means ± SDs.

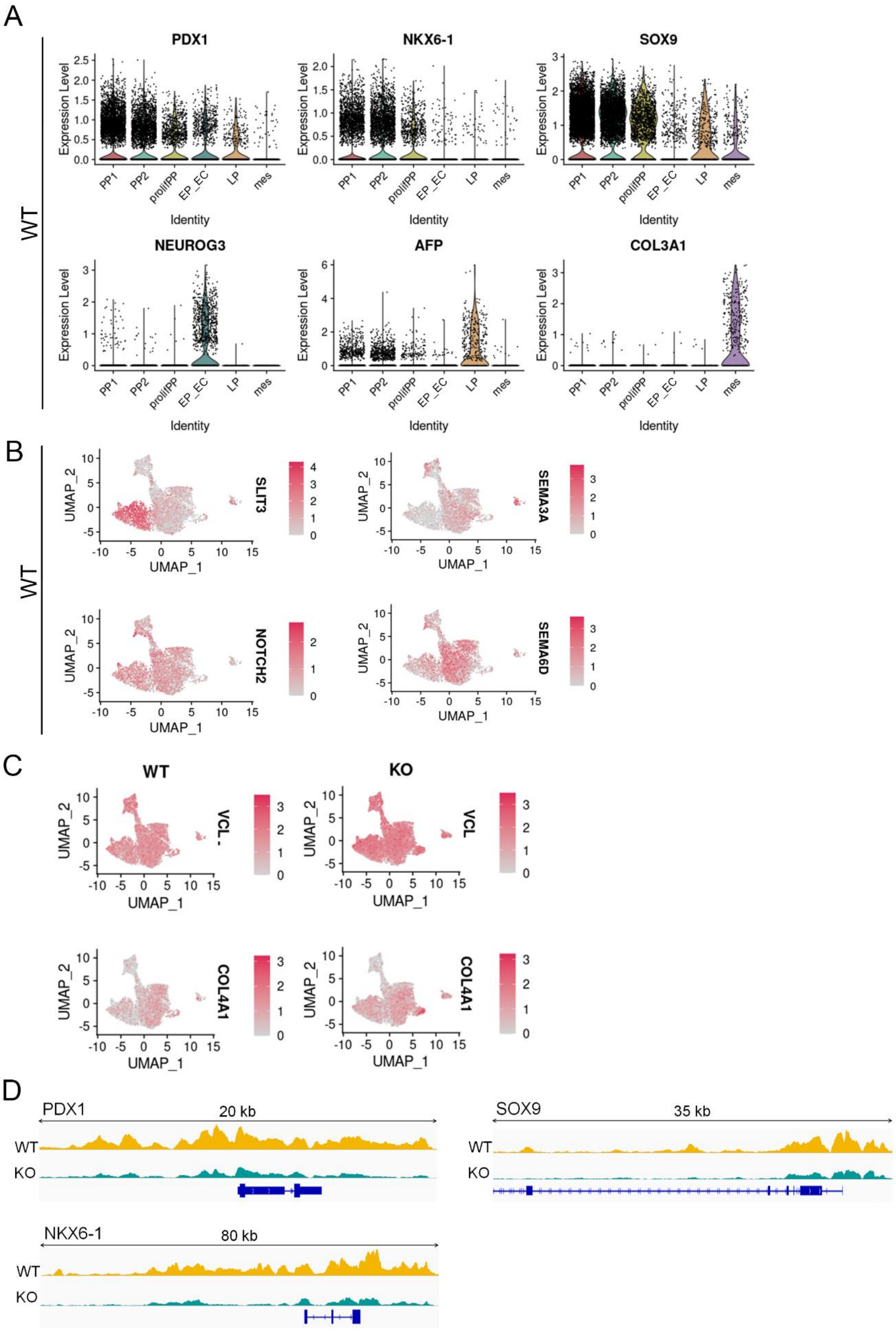
A
WT
PDX1
NKX6-1
SOX9
NEUROG3
AFP
COL3A1
Expression Level
Identity
PP1
PP2
prolifPP
EP_EC
LP
mes
B
WT
SLIT3
SEMA3A
NOTCH2
SEMA6D
UMAP_1
UMAP_2
C
WT
KO
VCL
COL4A1
D
PDX1
20 kb
SOX9
35 kb
NKX6-1
80 kb
WT
KO

Supplementary Figure 6. *ETV1* deletion disturbs *in vitro* differentiation towards pancreatic progenitors

A. Violon plots demonstrating the expression of well-known markers for each cluster in WT PPs: *PDX1*, *NKX6-1* and *SOX9* for pancreatic progenitors 1 (PP1), pancreatic progenitors 2 (PP2) and proliferating pancreatic progenitors (prolifPP); *NEUROG3* for endocrine progenitors and endocrine cells (EPandEC); *AFP* for liver progenitors (LP), *COL3A1* for mesenchyme cells (mes).

B. Feature plots showing expression (red) of *SLIT3*, *NRXN3*, *SEMA3A*, and *SEMA6D*.

C. Feature plots showing expression (red) of *VCL* and *COL4A1* in WT and KO cells.

D. ATAC-seq tracks demonstrating *PDX1*, *SOX9*, and *NKX6-1 loci* in WT (yellow) and KO (green) PPs. The peaks represent normalized and combined biological replicates.

Supplementary tables

Supplementary Table 1. Sequence of sgRNAs used for *ETV* genes targeting

**Clone name**	**sgRNA name**	**sgRNA sequence 5'->3'** (T7-**gRNA**-TracR)
**ETV1 KO, exon4**	ETV1 gRNA1 ex4	TAATACGACTCACTATAGGG**TCCCTTTGTAGAGTCAGCGT**TGTT<br>TTAGAGCTAGAAATAGCAAGTTAAAATAAGGCTAGTCCGTTAT<br>CAACTTGAAAAAGTGGCACCGAGTCGGTGCTTTT
	ETV1 gRNA2 ex4	TAATACGACTCACTATAGGG**ACGAGAAACCAAATGTCT**TGTT<br>TTAGAGCTAGAAATAGCAAGTTAAAATAAGGCTAGTCCGTTAT<br>CAA`CTTGAAAAAGTGGCACCGAGTCGGTGCTTTT
	ETV1 gRNA3 ex4	TAATACGACTCACTATAGGG**GGCTCATGATTCAGAAGGTG**TGTT<br>TTAGAGCTAGAAATAGCAAGTTAAAATAAGGCTAGTCCGTTAT<br>CAACTTGAAAAAGTGGCACCGAGTCGGTGCTTTT
**ETV1 KO-2, exon9**	ETV1 gRNA1 ex9	TAATACGACTCACTATAGGG**GGCATCGTCGGCAAAGG**TGTT<br>TTAGAGCTAGAAATAGCAAGTTAAAATAAGGCTAGTCCGTTAT<br>CAACTTGAAAAAGTGGCACCGAGTCGGTGCTTTT
	ETV1 gRNA2 ex9	TAATACGACTCACTATAGGG**TGCTTAAAGCCTTGTGG**TG<br>TTTTAGAGCTAGAAATAGCAAGTTAAAATAAGGCTAGTCCGTT<br>ATCAACTTGAAAAAGTGGCACCGAGTCGGTGCTTTT
	ETV1 gRNA3 ex9	TAATACGACTCACTATAGGG**GGCCGCACTGCCAACCA**TG<br>TTTTAGAGCTAGAAATAGCAAGTTAAAATAAGGCTAGTCCGTT<br>ATCAACTTGAAAAAGTGGCACCGAGTCGGTGCTTTT
**tKO**	ETV1 gRNA1 ex4	TAATACGACTCACTATAGGG**TCCCTTTGTAGAGTCAGCGT**TGTT<br>TTAGAGCTAGAAATAGCAAGTTAAAATAAGGCTAGTCCGTTAT<br>CAACTTGAAAAAGTGGCACCGAGTCGGTGCTTTT
	ETV1 gRNA2 ex4	TAATACGACTCACTATAGGG**ACGAGAAACCAAATGTCT**TGTT<br>TTAGAGCTAGAAATAGCAAGTTAAAATAAGGCTAGTCCGTTAT<br>CAACTTGAAAAAGTGGCACCGAGTCGGTGCTTTT
	ETV1 gRNA3 ex4	TAATACGACTCACTATAGGG**GGCTCATGATTCAGAAGGTG**TGTT<br>TTAGAGCTAGAAATAGCAAGTTAAAATAAGGCTAGTCCGTTAT<br>CAACTTGAAAAAGTGGCACCGAGTCGGTGCTTTT
	ETV4 gRNA1	TAATACGACTCACTATAGGG**CTCACGCTGCTGAAGGTGTA**TGTT<br>TTAGAGCTAGAAATAGCAAGTTAAAATAAGGCTAGTCCGTTAT<br>CAACTTGAAAAAGTGGCACCGAGTCGGTGCTTTT
	ETV4 gRNA2	TAATACGACTCACTATAGGG**AATGCCCGCGAGTCACCCTG**TGTT<br>TTAGAGCTAGAAATAGCAAGTTAAAATAAGGCTAGTCCGTTAT<br>CAACTTGAAAAAGTGGCACCGAGTCGGTGCTTTT
	ETV4 gRNA3	TAATACGACTCACTATAGGG**TCTTTACCTTCAGAGTCGAG**TGTT<br>TTAGAGCTAGAAATAGCAAGTTAAAATAAGGCTAGTCCGTTAT<br>CAACTTGAAAAAGTGGCACCGAGTCGGTGCTTTT
	ETV5 gRNA3	TAATACGACTCACTATAGGG**GATCTGGCTCACGATTCTGA**TGTT<br>TTAGAGCTAGAAATAGCAAGTTAAAATAAGGCTAGTCCGTTAT<br>CAACTTGAAAAAGTGGCACCGAGTCGGTGCTTTT
	ETV5 gRNA4	TAATACGACTCACTATAGGG**ATCTACACGCTACTGTCACT**TGTT<br>TTAGAGCTAGAAATAGCAAGTTAAAATAAGGCTAGTCCGTTAT<br>CAACTTGAAAAAGTGGCACCGAGTCGGTGCTTTT
	ETV5 gRNA5	TAATACGACTCACTATAGGG**ATCTACACGCTACTGTCACT**TGTT<br>TTAGAGCTAGAAATAGCAAGTTAAAATAAGGCTAGTCCGTTAT<br>CAACTTGAAAAAGTGGCACCGAGTCGGTGCTTTT

Supplmentary Table 2. Antibodies used for immunofluorescence staining

Antigen	Company	Catalog	Dilution	Primary / Secondary	Host	Reactivity
ETV1	Thermo Fisher Scientific	PA5-67447	1:100	Primary	Rabbit	Hu
ETV4	Santa Cruz	sc-166629	1:200	Primary	Mouse	Hu, Mus, Rat
ETV5	ProteinTech	13011-1-AP	1:100	Primary	Rabbit	Hu, Mus, Rat
OCT3/4	Santa Cruz	sc-5279	1:50	Primary	Mouse	Hu, Mus, Rat
NANOG	R&D	AF1997	1:40	Primary	Goat	Hu
ITGA5	Santa Cruz	sc-9969	1:100	Primary	Mouse	Hu
PXN	Santa Cruz	sc-365379	1:100	Primary	Mouse	Hu, Mus, Rat
VCL	Santa Cruz	sc-73614	1:100	Primary	Mouse	Hu, Mus, Rat
CDH1	R&D	AF748	1:100	Primary	Goat	Hu, Mus
F-ACTIN	Invitrogen	A12379	1:200	Primary-conjugated AF488	–	Hu
BRA	R&D	MAB20851	1:400	Primary	Rabbit	Hu
ISL1	DSHB	39.4D-5	1:100	Primary	Mouse	Hu, Mus, Rat
SOX2	Cell Signaling Technology	23064s	1:200	Primary	Rabbit	Hu, Mus
SOX17	R&D	AF1924	1:100	Primary	Goat	Hu
FOXA2	R&D	AF2400	1:100	Primary	Goat	Hu
PDX1	R&D	AF2419	1:100	Primary	Goat	Hu
NKX6-1	DSHB	F55A10	1:20	Primary	Mouse	Hu, Mus
CHGA	Abcam	ab15160	1:200	Primary	Rabbit	Hu
COL4A	Santa Cruz	sc-59814	1:100	Primary	Mouse	Hu

AlexaFluor 488	Jackson ImmunoResearch	715-545-147	1:400	Secondary	Donkey	anti-Goat IgG (H+L)
AlexaFluor 488	Jackson ImmunoResearch	711-545-152	1:400	Secondary	Donkey	anti-Rabbit IgG (H+L)
AlexaFluor 488	Jackson ImmunoResearch	705-545-150	1:400	Secondary	Donkey	anti-Mouse IgG (H+L)
TRITC	Jackson ImmunoResearch	705-025-147	1:400	Secondary	Donkey	anti-Goat IgG (H+L)
TRITC	Jackson ImmunorResearch	715-025-150	1:400	Secondary	Donkey	anti-Mouse IgG (H+L)
TRITC	Jackson ImmunoResearch	711-025-152	1:400	Secondary	Donkey	anti-Rabbit IgG (H+L)
AlexaFluor 647	Jackson ImmunoResearch	705-605-147	1:400	Secondary	Donkey	anti-Goat IgG (H+L)
AlexaFluor 647	Jackson ImmunoResearch	711-605-152	1:400	Secondary	Donkey	anti-Rabbit IgG (H+L)
AlexaFluor 647	Jackson ImmunoResearch	715-605-151	1:400	Secondary	Donkey	anti-Mouse IgG (H+L)

Hu – human, Mus – mouse

Supplementary Table 3. Antibodies used for western blotting

Antibody name	Company	Catalog	Dilution	Diluted in	Incubation time
ETV1	R&D	MAB9389	1:500	5% non-fat dry milk	O/N
ETV1*	Invitrogen	PA5-67447	1:1000	3% BSA in TBS-T	O/N
ETV4	Santa Cruz	sc-166629	1:1000	5% non-fat dry milk	O/N
ETV5	ProteinTech	13011-1-AP	1:1000	5% non-fat dry milk	O/N
pHH3*	Millipore	06-570	1:1000	3% BSA in TBS-T	O/N
Phospho-AKT (Ser473)	Cell Signaling	9271	1:1000	1% BSA in TBS-T	O/N
VCL	Santa Cruz	sc-73614	1:1000	3% BSA in TBS-T	O/N
Total AKT	Cell Signaling	4691	1:1000	0.125% non-fat dry milk	O/N
GAPDH	Millipore	MAB374	1:2000	0.125% non-fat dry milk	O/N

Antibody name	Company	Catalog	Dilution	Diluted in	Incubation time
ETV1	R&D	MAB9389	1:500	5% non-fat dry milk	O/N
ETV1*	Invitrogen	PA5-67447	1:1000	3% BSA in TBS-T	O/N
ETV4	Santa Cruz	sc-166629	1:1000	5% non-fat dry milk	O/N
ETV5	ProteinTech	13011-1-AP	1:1000	5% non-fat dry milk	O/N
pHH3*	Millipore	06-570	1:1000	3% BSA in TBS-T	O/N
Phospho-AKT (Ser473)	Cell Signaling	9271	1:1000	1% BSA in TBS-T	O/N
VCL	Santa Cruz	sc-73614	1:1000	3% BSA in TBS-T	O/N
Total AKT	Cell Signaling	4691	1:1000	0.125% non-fat dry milk	O/N
GAPDH*	Millipore	MAB374	1:5000	3% BSA in TBS-T	O/N
anti-rabbit	Sigma Aldrich	A9169	1:20000	0.125% non-fat dry milk	1 h
anti-mouse	Sigma Aldrich	A9044	1:20000	0.125% non-fat dry milk	1 h

O/N=overnight

*subcellular fractioning

Supplementary Table 4. Antibodies used for flow cytometry analysis

Antigen	Company	Catalog	Dilution	Primary/ Secondary	Host	Reactivity
CDH1	R&D	AF748	1:100	Primary	Goat	Hu, Mus
pHH3	Millipore	06-570	1:300	Primary	Rabbit	Hu, Mus
Ki67	BD Pharmingen	556027	1:300	Primary	Mouse	Hu, Mus, Rat
KLF4	R&D	AF3640	1:300	Primary	Goat	Hu
SOX2	R&D	MAB2018	1:300	Primary	Mouse	Hu, Mu, Rat

OCT3/4	Santa Cruz	sc-5279	1:300	Primary	Mouse	Hu, Mus, Rat
NANOG	R&D	AF1997	1:200	Primary	Goat	Hu
ETV1	Thermo Fisher Scientific	PA5-67447	1:300	Primary	Rabbit	Hu
ETV4	Santa Cruz	sc-166629	1:600	Primary	Mouse	Hu, Mus, Rat
ETV5	ProteinTech	13011-1-AP	1:300	Primary	Rabbit	Hu, Mus, Rat
ITGA5	Santa Cruz	sc-9969	1:100	Primary	Mouse	Hu
PDX1	R&D	AF2419	1:100	Primary	Goat	Hu
NKX6-1	DSHB	F55A10	1:20	Primary	Mouse	Hu, Mus
AlexaFluor 488	Jackson ImmunoResearch	715-545-147	1:400	Secondary	Donkey	anti-Goat IgG (H+L)
AlexaFluor 488	Jackson ImmunoResearch	711-545-152	1:400	Secondary	Donkey	anti-Rabbit IgG (H+L)
AlexaFluor 488	Jackson ImmunoResearch	705-545-150	1:400	Secondary	Donkey	anti-Mouse IgG (H+L)
TRITC	Jackson ImmunoResearch	705-025-147	1:400	Secondary	Donkey	anti-Goat IgG (H+L)
TRITC	Jackson ImmunoResearch	715-025-150	1:400	Secondary	Donkey	anti-Mouse IgG (H+L)
TRITC	Jackson ImmunoResearch	711-025-152	1:400	Secondary	Donkey	anti-Rabbit IgG (H+L)
AlexaFluor 647	Jackson ImmunoResearch	705-605-147	1:400	Secondary	Donkey	anti-Goat IgG (H+L)
AlexaFluor 647	Jackson ImmunoResearch	711-605-152	1:400	Secondary	Donkey	anti-Rabbit IgG (H+L)
AlexaFluor 647	Jackson ImmunoResearch	715-605-151	1:400	Secondary	Donkey	anti-Mouse IgG (H+L)

Hu – human, Mus – mouse

Supplementary Table 5. Sequences of qPCR primers

Primer name	Primer sequence (5'-3')
ACTB_F	ACAGAGCCTCGCCTTTGCCGAT
ACTB_R	ATCATCCATGGTGAGCTGGCGG
ChIP_CDKN1A_F	TGGAGATCAGGTTGCCCTTT
ChIP_CDKN1A_R	TCTGGCCTCAAGATGCTTTGT
ChIP_COL4A6_F	GAGGGAGTGACGCTCAGTTA
ChIP_COL4A6_R	TCTCATTGGTGTGCGTACCA
ChIP_ITGA5_F	TGAATGACCCCAAACTCCTGG
ChIP_ITGA5_R	CGAACGCTGTCTCTGTACCT
ChIP_JUN_F	ACCTTAAGGGCGGTATTCCC
ChIP_JUN_R	GGAAACAACAACCACCCCTC
ChIP_PDGFRB_F	TGTGCCAATTCACCCCTGG
ChIP_PDGFRB_R	AAATGCACTCTCAGCCTCCTG
ChIP_VCL_F	GCTCCCACCATGCCCTTAT
ChIP_VCL_R	GCATCTCGAAAAGGGACCAGT
ETV4_F	GATGGAGCGGAGGATGAAAG
ETV4_R	GGGCTGTGGAAAGCTAGGTT
ETV5_F	ATGGACGGGTTTTATGATCAGCAAGT
ETV5_R	TTAGCACCAAGAGCCTGCTC
GAPDH_F	AAGGTGAAGGTCGGAGTCAA
GAPDH_R	AATGAAGGGGTCATTGATGG
GATA4_F	CGACACCCCAATCTCGATATGT
GATA4_R	ACAGATAGTGACCCGTCCCA
HHEX_F	GAAATATCTCTCTCCGCCCGA
HHEX_R	GGGTTCTCCTGTTTTAGTCTCC
HOXA1_F	GGTGTCCTACTCCCACTCAAG
HOXA1_R	TCTCCAACTTTCCCTGTTTTGG
MESP1_F	CACCGTCCCCGCTCCTT
MESP1_R	AGAGACGGCGTCAGTTGTCC
MIXL1_F	GGATCCAGCTTTTATTTTCTCCCC
MIXL1_R	TCCAGGAGCACAGTGGTTGA
OTX2_F	CGAGGGTGCAGGTATGGTTTA
OTX2_R	GCCACTTGTTCCACTCTCTGA
PAX6_F	CGATAACATACCAAGCGTGTCA
PAX6_R	TGCCCGTTCAACATCCTTAGT
PDGFRA_F	CTATGTGCCAGACCCAGATGT
PDGFRA_R	CAGGAGTCTCGGGATCAGTTG
SOX1_F	GCAGGTCCAAGCACTTACAAG
SOX1_R	GGGTGGTGGTGGTAATCTCTT
SOX17_F	CGCTTTCATGGTGTGGGCTA
SOX17_R	CTTCCACGACTTGCCCAGC

SOX2_F	CATGCACCGCTACGACGT
SOX2_R	CTGCGAGTAGGACATGCTGTA
SOX7_F	TTTGGGCCAAGGACGAGAGG
SOX7_R	CTTCCACGACTTTCCCAGCAT
MNX1_F	CAAGCTCAACAAGTACCTGTCG
MNX1_R	GCTGCGTTTCCATTTCATCCG
CASZ1_F	TGGAAAGTCACCCAAAGCCG
CASZ1_R	CCCAAACTCTCGTAGCAGGT
MEIS2_F	TCCAGCATCTCACACATCCG
MEIS2_R	ACTGGTCAATCATGGGCTGT
MYH6_F	CTACGCAACTGCCGATACTG
MYH6_R	TGAGATTTTCCCGGTGGAGAG
TNNT2_F	GAATGAGCGGGAGAAGGAGC
TNNT2_R	TGCTTCTGGATGTAACCCCC

Supplementary References

1. Yiangou, L. et al. Method to Synchronize Cell Cycle of Human Pluripotent Stem Cells without Affecting Their Fundamental Characteristics. *Stem Cell Rep* **12**, 165-179 (2019).

Gating Strategy for Flow Cytometry

Example 1

1. Live cells

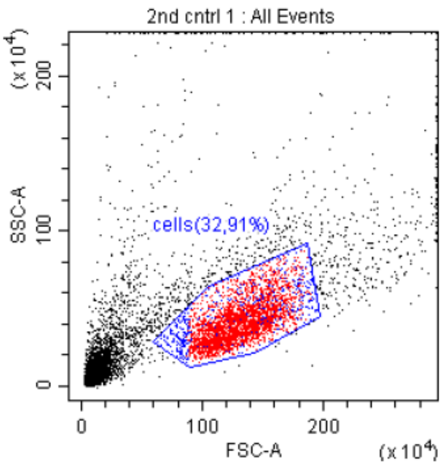

2. Singlets

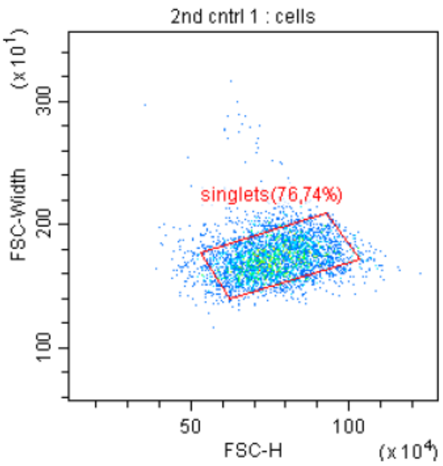

3. Gating for fluorescence signal – on 2nd control, singlets

2nd antibody control for AF647 on singlets

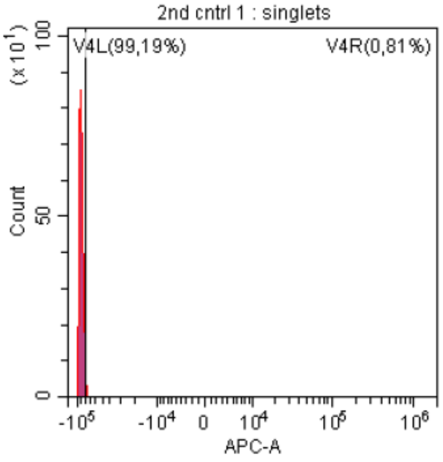

2nd antibody control for AF488 on singlets

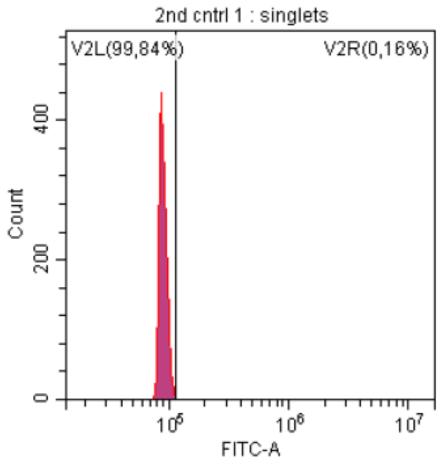

Example 2

1. Live cells

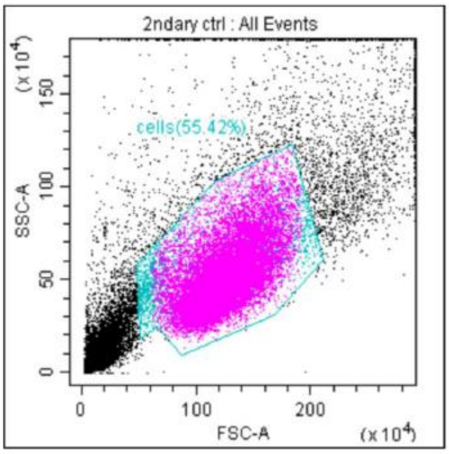

2. Singlets

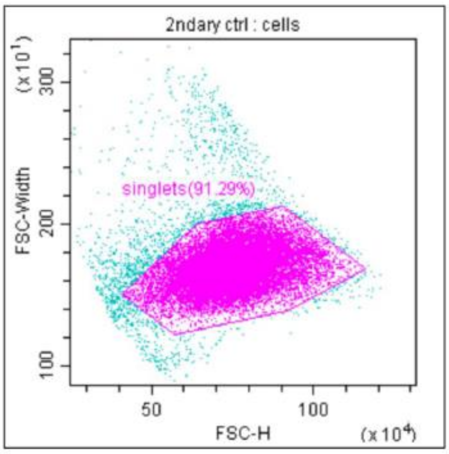

3. Gating for fluorescence signal – on 2nd control, singlets

2nd antibody control for AF647 on singlets

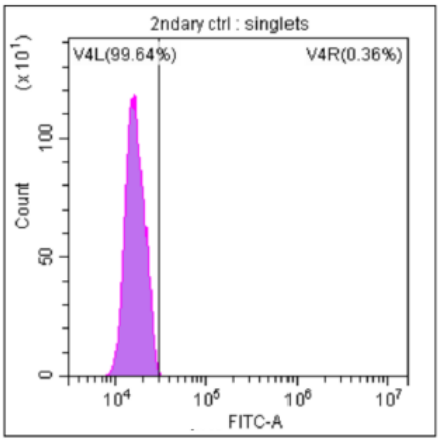

2nd antibody control for AF488 on singlets

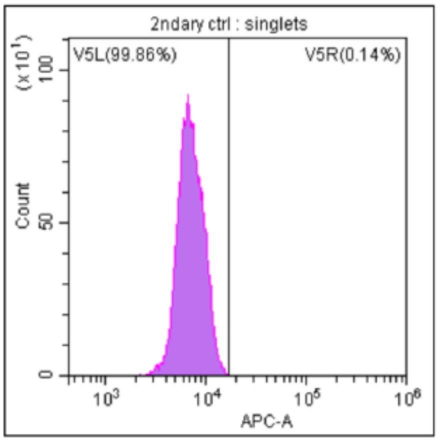

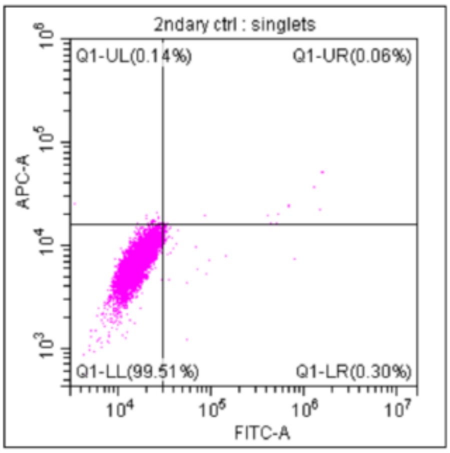

Supplement: Supplementary file 1 — Supplementary information [file 41467_2025_56591_MOESM1_ESM.pdf]
