# Supplementary material for: ETVs dictate hPSC differentiation by tuning biophysical properties
Source: Nat Commun. 2025 Feb 26;16:1999. doi: 10.1038/s41467-025-56591-6 (PMC11865489; doi:10.1038/s41467-025-56591-6)
Supplement: Supplementary file 3 — Description of Additional Supplementary Files [file 41467_2025_56591_MOESM3_ESM.pdf]

Description of Additional Supplementary Files

Supplementary Data 1: RNA-seq results for KO vs. WT, tKO vs. WT, and tKO vs. KO comparisons. The analysis was performed with DESeq2 package, with the use of the Wald test for significance difference testing. The p values are adjusted for multiple tests using the Benjamini and Hochberg procedure. Differentially expressed genes (DEGs) were analyzed for biological term enrichment analysis in DAVID online software, using the Fisher Exact statistics to list annotation terms and their associated genes under study. The p values are adjusted for multiple comparisons using Benjamini and Hochberg approach. The results from that analysis are in separate sheets: KOvsWT_Upregulated, KOvsWT_Downregulated, tKOvsWT_Upregulated, tKOvsWT_Downregulated, tKOvsKO_Upregulated, tKOvsKO_Downregulated
