# Supplementary material for: ETVs dictate hPSC differentiation by tuning biophysical properties
Source: Nat Commun. 2025 Feb 26;16:1999. doi: 10.1038/s41467-025-56591-6 (PMC11865489; doi:10.1038/s41467-025-56591-6)
Supplement: Supplementary file 5 — Reporting Summary [file 41467_2025_56591_MOESM5_ESM.pdf]

nature portfolio

Corresponding author(s): Borowiak Malgorzata

Last updated by author(s): 01/07/2025

Reporting Summary

Nature Portfolio wishes to improve the reproducibility of the work that we publish. This form provides structure for consistency and transparency in reporting. For further information on Nature Portfolio policies, see our Editorial Policies and the Editorial Policy Checklist.

Statistics

For all statistical analyses, confirm that the following items are present in the figure legend, table legend, main text, or Methods section.

n/a	Confirmed	
☐	☒	The exact sample size (*n*) for each experimental group/condition, given as a discrete number and unit of measurement
☐	☒	A statement on whether measurements were taken from distinct samples or whether the same sample was measured repeatedly
☐	☒	The statistical test(s) used AND whether they are one- or two-sided<br>*Only common tests should be described solely by name; describe more complex techniques in the Methods section.*
☐	☒	A description of all covariates tested
☐	☒	A description of any assumptions or corrections, such as tests of normality and adjustment for multiple comparisons
☐	☒	A full description of the statistical parameters including central tendency (e.g. means) or other basic estimates (e.g. regression coefficient) AND variation (e.g. standard deviation) or associated estimates of uncertainty (e.g. confidence intervals)
☒	☐	For null hypothesis testing, the test statistic (e.g. *F*, *t*, *r*) with confidence intervals, effect sizes, degrees of freedom and *P* value noted<br>*Give P values as exact values whenever suitable.*
☒	☐	For Bayesian analysis, information on the choice of priors and Markov chain Monte Carlo settings
☒	☐	For hierarchical and complex designs, identification of the appropriate level for tests and full reporting of outcomes
☒	☐	Estimates of effect sizes (e.g. Cohen's *d*, Pearson's *r*), indicating how they were calculated

Our web collection on statistics for biologists contains articles on many of the points above.

Software and code

Policy information about availability of computer code

Data collection	Flow cytometry data were acquired using a CytoFLEX Flow (Beckman Coulter, USA), or Guava EasyCyte HT (Millipore, USA), or NovoCyte Flow Cytometer (Agilent Technologies, USA).<br>Photomicrographs of live cells were taken with the IncuCyte Base Analysis Software (Sartorius, Germany).<br>Crystal violet analysis was performed using TECAN Sparc (Tecan, Switzerland).<br>Images were obtained as follows: brightfield images using Leica DM IL-Led (Leica, Germany), fluorescence images using Nikon A1Rsi (Nikon, Germany), super-resolution microscopy was performed with a ZEISS Elyra 7 with Lattice SIM (Zeiss, Germany) microscope, with ZEN Black 3.1 software (for imaging).<br>hPSC 3D spheres were analyzed with W8 Physical Cytometer (Cell Dynamics, Italy).
Data analysis	The analyses were conducted as described in the manuscript.<br>Bulk RNA-seq analysis: adapter trimming using fastp v0.12.4, alignment using STAR v2.7.10b, generating bams using samtools v1.10, raw count using featureCounts v1.6.3, obtaining DEGs with iDEP using DESeq2 v9.4 (http://bioinformatics.sdstate.edu/idep94/), graphs and statistical analysis using Excel, R and/or GraphPad Prism v8.4.2, KEGG, GO, and WikiPathway analysis using GeneCodis v4 (https://genecodis.genyo.es/), in silico motif analysis using Pscan v1.6 (http://159.149.160.88/pscan/).<br>ScRNA-seq analysis: to align reads, generate feature-barcode matrices, aggregates outputs from multiple runs CellRanger v8.0 was used, to perform clustering and other secondary analysis Seurat Package v4.3.0 was used.<br>ATAC-seq analysis: initial analysis, peak calling, annotation, and generating BigWig output using nfcore/atacseq bioinformatic pipeline v2.1.2 (https://nf-co.re/atacseq/2.1.2/), analysis of the differential accessibility regions using DESeq2 v1.6.3, motif enrichment using HOMER v4.11.<br><br>All immunostaining, immunoblotting and RT-qPCR experiments were performed in biological replicates (N ≥3), with individual data points depicted in the graphs plotted by GraphPad Prism v8.4.2 for Windows (GraphPad Software, Boston, MA USA). For statistical analyses,

unpaired two-tailed Student t tests or one-way ANOVA for multiple comparisons were performed. All values are presented as means ± SDs and the p-values are shown on individual figures. The number of replicates of the experiment (N) are described in the relevant figure legend.

Flow cytometry data analysis, including gating, quantification, and the generation of density plots/histograms, was performed with FlowJo Software v10 (BD, USA) or NovoExpress Software v1.6.2 (Agilent Technologies, USA).

Imaging analysis: ZEN Blue 3.1 software (Zeiss, Germany), NIS Viewer 5.21 software. For quantification of the immunofluorescence signal derived from the images ImageJ v1.54f (LOCI, University of Wisconsin, USA) was used.

For manuscripts utilizing custom algorithms or software that are central to the research but not yet described in published literature, software must be made available to editors and reviewers. We strongly encourage code deposition in a community repository (e.g. GitHub). See the Nature Portfolio guidelines for submitting code & software for further information.

Data

Policy information about availability of data

All manuscripts must include a data availability statement. This statement should provide the following information, where applicable:

- Accession codes, unique identifiers, or web links for publicly available datasets
- A description of any restrictions on data availability
- For clinical datasets or third party data, please ensure that the statement adheres to our policy

The data generated in this study are provided in the Source Data file. Raw RNA-seq, scRNA-seq, and ATAC-seq data generated during the study have been deposited in the NCBI GEO database under accession number GSE227794. Raw image files are available from the corresponding author upon a reasonable request. The authors declare that cells are available for the research community upon a request, from the corresponding author.

Research involving human participants, their data, or biological material

Policy information about studies with human participants or human data. See also policy information about sex, gender (identity/presentation), and sexual orientation and race, ethnicity and racism.

Reporting on sex and gender	The manuscript does not contain data involving human participants.
Reporting on race, ethnicity, or other socially relevant groupings	The manuscript does not contain data involving human participants.
Population characteristics	The manuscript does not contain data involving human participants.
Recruitment	The manuscript does not contain data involving human participants.
Ethics oversight	The manuscript reports on expression studies with human fetal pancreas. Tissue was obtained under IRB approval IRB-3097 to M.B., and was processed at Baylor College of Medicine, Houston, TX, USA

Note that full information on the approval of the study protocol must also be provided in the manuscript.

Field-specific reporting

Please select the one below that is the best fit for your research. If you are not sure, read the appropriate sections before making your selection.

☒ Life sciences ☐ Behavioural & social sciences ☐ Ecological, evolutionary & environmental sciences

For a reference copy of the document with all sections, see nature.com/documents/nr-reporting-summary-flat.pdf

Life sciences study design

All studies must disclose on these points even when the disclosure is negative.

Sample size	Sample size was determined based on preliminary experiments and POWER calculations.
Data exclusions	None data were excluded from analysis.
Replication	Each experiments was performed in replicates, all replicates reproduced the results/observations.
Randomization	Samples were allocated randomly.
Blinding	IF signal was quantified in blinded manner by two independent researchers.

Reporting for specific materials, systems and methods

We require information from authors about some types of materials, experimental systems and methods used in many studies. Here, indicate whether each material, system or method listed is relevant to your study. If you are not sure if a list item applies to your research, read the appropriate section before selecting a response.

Materials & experimental systems

n/a	Involved in the study
☐	☒ Antibodies
☐	☒ Eukaryotic cell lines
☒	☐ Palaeontology and archaeology
☒	☐ Animals and other organisms
☒	☐ Clinical data
☒	☐ Dual use research of concern
☒	☐ Plants

Methods

n/a	Involved in the study
☒	☐ ChIP-seq
☐	☒ Flow cytometry
☒	☐ MRI-based neuroimaging

Antibodies

Antibodies used

Primary antibodies used for immunofluorescence staining (IF) and/or flow cytometry (FC): anti-ETV1 antibody (IF, 1:100 and FC, 1:300, cat. #PA5-67447, Thermo Fisher Scientific), anti-ETV4 antibody (IF, 1:200 and FC, 1:600, cat. #sc-166629, Santa Cruz), anti-ETV5 antibody (IF, 1:100 and FC, 1:300, cat. #13011-1-AP, ProteinTech), anti-OCT3/4 antibody (IF, 1:50 and FC, 1:300, cat. #sc-5279, Santa Cruz), anti-NANOG antibody (IF, 1:40 and FC, 1:300, cat. #AF1997, R&D), anti-ITGA5 antibody (IF/FC, 1:100, cat. #sc-9969, Santa Cruz), anti-PXN antibody (IF, 1:100, cat. #sc-365379, Santa Cruz), anti-VCL antibody (IF, 1:100, cat. #sc-73614, Santa Cruz), anti-CDH1 antibody (IF/FC, 1:100, cat. #AF748, R&D), anti-F-ACTIN-AF488 conjugated primary antibody (IF, 1:200, cat. #A12379, Invitrogen), anti-BRA antibody (IF, 1:400, cat. #MAB20851, R&D), anti-ISL1 antibody (IF, 1:100, cat. #39.4D-5, DSHB), anti-SOX2 antibody (IF, 1:200, cat. #23064s, Cell Signaling Technology; FC, 1:300, cat. #MAB2018, R&D), anti-SOX17 antibody (IF, 1:100, cat. #AF1924, R&D), anti-FOXA2 antibody (IF, 1:100, cat. #AF2400, R&D), anti-PDX1 antibody (IF/FC, 1:100, cat. #AF2419, R&D), anti-NKX6-1 antibody (IF/FC, 1:20, cat. #F55A10, DSHB), anti-CHGA antibody (IF, 1:200, cat. #ab15160, Abcam), anti-COL4A antibody (IF, 1:100, cat. #sc-59814, Santa Cruz), anti-pHH3 antibody (FC, 1:300, cat. #06-570, Millipore), anti-Ki76 antibody (FC, 1:300, cat. #556027, BD Pharmingen), anti-KLF4 antibody (FC, 1:300, cat. #AF3640, R&D).

Secondary antibodies used for immunofluorescence staining (IF) and/or flow cytometry (FC) were purchased from Jackson ImmunoResearch (JIR): AlexaFluor488 Donkey anti-Goat IgG (H+L) antibody (1:400, cat. #715-545-147), AlexaFluor488 Donkey anti-Rabbit IgG (H+L) antibody (1:400, cat. #711-545-152), AlexaFluor488 Donkey anti-Mouse IgG (H+L) antibody (1:400, cat. #705-545-150), TRITC Donkey anti-Goat IgG (H+L) antibody (1:400, cat. #705-025-147), TRITC Donkey anti-Mouse IgG (H+L) antibody (1:400, cat. #715-025-150), TRITC Donkey anti-Rabbit IgG (H+L) antibody (1:400, cat. #711-025-152), AlexaFluor647 Donkey anti-Goat IgG (H+L) antibody (1:400, cat. #705-605-147), AlexaFluor647 Donkey anti-Rabbit IgG (H+L) antibody (1:400, cat. #711-605-152), AlexaFluor647 Donkey anti-Mouse IgG (H+L) antibody (1:400, cat. #715-605-151).

Antibodies used for western blotting: anti-ETV1 antibody (1:500, cat. #MAB9389, R&D or 1:1000, cat. #PA5-67447, Invitrogen), anti-ETV4 antibody (1:1000, cat. #sc-166629, Santa Cruz), anti-ETV5 antibody (1:1000, cat. #sc-13011-1-AP, ProteinTech), anti-phospho-AKT (Ser473) antibody (1:1000, cat. #9271, Cell Signaling), anti-VCL antibody (1:1000, cat. #sc-73614, Santa Cruz), anti-total AKT antibody (1:1000, cat. #4691, Cell Signaling), anti-pHH3 antibody (1:1000, cat. #06-570, Millipore), anti-GAPDH antibody (1:2000 or 1:5000, cat. #MAB374, Millipore), anti-rabbit antibody (1:20000, cat. #A9169, Sigma Aldrich), anti-mouse antibody (1:20000, cat. #A9044, Sigma Aldrich).

Validation

All antibodies were purchased from verified well-known, commonly used suppliers. Information about the validation of individual antibodies is available on the manufacturer's websites. When applicable the antibodies were verified on human tissue samples and/or KO cell lines. Negative control (none antibodies, secondary antibodies only) were routinely used.

Eukaryotic cell lines

Policy information about cell lines and Sex and Gender in Research

Cell line source(s)

HUES8 hESC-Harvard University (HVRD), USA; H1 hESC-WiCell Stem Cell Bank, USA; ESI017 hPSC-ESIBIO, Singapore.

Authentication

The listed cells are commonly used in the laboratories, which conducted research described in the manuscript. KO lines were routinely tested for desired genotype. Karyotyping was performed every 12 months.

Mycoplasma contamination

Cells were tested every two weeks for Mycoplasma contamination and found negative.

Commonly misidentified lines
(See ICLAC register)

No commonly misidentified cell lines were utilized in this study.

Plants

Seed stocks

The manuscript does not contain research concerning plants.

Novel plant genotypes

The manuscript does not contain research concerning plants.

Authentication

The manuscript does not contain research concerning plants.

Flow Cytometry

Plots

Confirm that:

☒ The axis labels state the marker and fluorochrome used (e.g. CD4-FITC).

☒ The axis scales are clearly visible. Include numbers along axes only for bottom left plot of group (a 'group' is an analysis of identical markers).

☒ All plots are contour plots with outliers or pseudocolor plots.

☒ A numerical value for number of cells or percentage (with statistics) is provided.

Methodology

Sample preparation

Detailed protocol for Flow Cytometry is described in the Methods section. Briefly, hPSCs or PPs were fixed with 4% PFA-0.1% Saponin-1% BSA. To obtain single-cell suspension hPSCs and PPs were incubated with Tryple or 1% Trypsin respectively. The staining procedure was performed on ice. Cells were stained with primary antibody O/N in 4 °C respectively to the experiment. The next day, cells were stained with a secondary antibody. Table 4 in the Methods section lists all primary and secondary antibodies. For analysis, cells were resuspended in 1x PBS.

Instrument

CytoFLEX S Flow Cytometer (Beckman Coulter, USA), or Guava EasyCyte HT (Millipore, USA), or NovoCyte Flow Cytometer (Agilent Technologies, USA).

Software

The data were analyzed using FlowJo software v10.

Cell population abundance

Cell sorting was not conducted.

Gating strategy

First, live cells were identified using plot SSC-Area/FSC-Area (gate live cells). Next, the singlets were identified based on plot FCS-Width/FSC-Height (gate singlets). The boundaries between the "positive" and "negative" signals were established using secondary antibody control cells (cells stained only for secondary antibody) based on histogram gated on singlets.

☒ Tick this box to confirm that a figure exemplifying the gating strategy is provided in the Supplementary Information.
